# Measuring the effects of listening for leisure on outcome after stroke (MELLO): A pilot randomized controlled trial of mindful music listening

**DOI:** 10.1177/1747493019841250

**Published:** 2019-04-02

**Authors:** Satu Baylan, Caroline Haig, Maxine MacDonald, Ciara Stiles, Jake Easto, Meigan Thomson, Breda Cullen, Terence J Quinn, David Stott, Stewart W Mercer, Niall M Broomfield, Heather Murray, Jonathan J Evans

**Affiliations:** 1 Institute of Health and Wellbeing, University of Glasgow, Glasgow, UK; 2 Robertson Centre for Biostatistics, University of Glasgow, Glasgow, UK; 3 Stroke Psychology Service, NHS Greater Glasgow and Clyde, Glasgow, UK; 4 Institute of Cardiovascular and Medical Sciences, University of Glasgow, Glasgow, UK; 5 Usher institute of population health sciences and informatics, University of Edinburgh, Edinburgh, UK; 6 Norwich Medical School, University of East Anglia, England, UK

**Keywords:** Mindfulness, music, mood, cognition, rehabilitation, stroke, ischemic, audiobooks

## Abstract

**Background:**

Cognitive deficits and low mood are common post-stroke. Music listening is suggested to have beneficial effects on cognition, while mindfulness may improve mood. Combining these approaches may enhance cognitive recovery and improve mood early post-stroke.

**Aims:**

To assess the feasibility and acceptability of a novel mindful music listening intervention.

**Methods:**

A parallel group randomized controlled feasibility trial with ischemic stroke patients, comparing three groups; mindful music listening, music listening and audiobook listening (control group), eight weeks intervention. Feasibility was measured using adherence to protocol and questionnaires. Cognition (including measures of verbal memory and attention) and mood (Hospital Anxiety and Depression Scale) were assessed at baseline, end of intervention and at six-months post-stroke.

**Results:**

Seventy-two participants were randomized to mindful music listening (*n* = 23), music listening (*n* = 24), or audiobook listening (*n* = 25). Feasibility and acceptability measures were encouraging: 94% fully consistent with protocol; 68.1% completing ≥6/8 treatment visits; 80–107% listening adherence; 83% retention to six-month endpoint. Treatment effect sizes for cognition at six month follow-up ranged from d = 0.00 ([−0.64,0.64], music alone), d = 0.31, ([0.36,0.97], mindful music) for list learning; to d = 0.58 ([0.06,1.11], music alone), d = 0.51 ([−0.07,1.09], mindful music) for immediate story recall; and d = 0.67 ([0.12,1.22], music alone), d = 0.77 ([0.16,1.38]mindful music) for attentional switching compared to audiobooks. No signal of change was seen for mood. A definitive study would require 306 participants to detect a clinically substantial difference in improvement (z-score difference = 0.66, *p* = 0.017, 80% power) in verbal memory (delayed story recall).

**Conclusions:**

Mindful music listening is feasible and acceptable post-stroke. Music listening interventions appear to be a promising approach to improving recovery from stroke.

## Introduction

Cognitive deficits, particularly in the domains of attention, executive function and verbal memory,^1^ and emotional problems such as depression and anxiety^2^ are common post-stroke.

The potential benefits of music in the treatment of mental disorders are long recognized.^3^ Music activates brain areas related to attention, memory, motor and affective processing^4^ and has potential as a safe, accessible, and low-cost intervention to alleviate and/or prevent psychological morbidity after stroke.^5,6^

Regular, active music listening (i.e. consciously attending to music rather than simply listening to music in the background whilst doing other activities) may also induce relaxation, positive mood change, and evoke memories and reflective thoughts about the past and the future.^7,8^ A parallel can be drawn with mindfulness training, which has been shown to reduce depressive symptoms and to improve performance on measures of working memory and sustained attention in healthy individuals.^9^ Mindfulness aims to promote wellbeing through learning to pay attention to the present moment in a non-judgemental way. Mindfulness has also been reported to enhance mood following brain injury^10^ and has potential applications after stroke.^11^

It is possible that music listening and mindfulness share a common mechanism of action via attentional control,^12^ through which they operate by reducing self-focus and increasing metacognitive control and attention to present experiences thus limiting rumination on negative thoughts and feelings. The effects of music listening may therefore be enhanced by incorporating components of mindfulness – music listening could be a vehicle by which attentional control skills associated with mindfulness practice are developed.

We performed a pilot feasibility randomized controlled trial (RCT) of a novel intervention combining music listening with brief mindfulness practice aimed at reducing low mood and promoting cognitive recovery in the initial months post-stroke.

## Aims

To assess the feasibility and acceptability of incorporating brief mindfulness training into a music listening intervention in an RCT context post-stroke.

Secondary aims

- to determine recruitment and retention rates over 18 months,
- to determine estimated effect sizes (with 95% confidence intervals) for change on key outcome measures of mood and cognition.

## Methods

**Figure 1.**
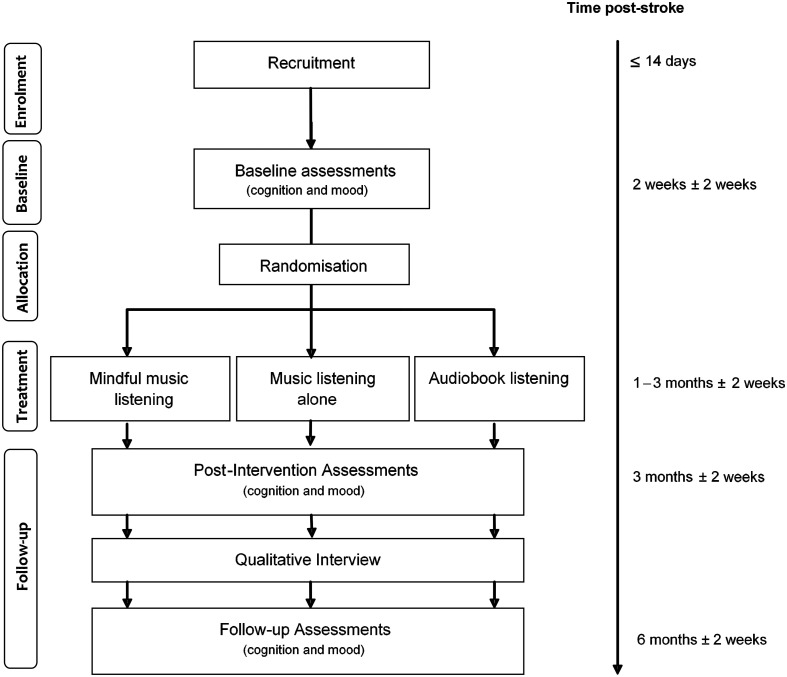
Participant flow.

A three-arm, parallel group, single-blind pilot RCT comparing (1) music listening with brief mindfulness training (referred to as mindful-music listening from here on), (2) music listening alone, and (3) audiobook listening (control arm). During the study, all participants received treatment as usual, including multidisciplinary rehabilitation. Figure 1 shows participant flow through the study. Reporting follows CONSORT extension for pilot and feasibility trials.^13^ The study was approved by the West of Scotland Research Ethics Committee (14/WS/1089). All participants gave written informed consent. The study was registered with clinicaltrials.gov (NCT02259062) and the UK Clinical Research Network (ID 18019).

### Participants

#### Eligibility criteria

Native English-speaking adults (aged ≥ 18, upper age limit of 80 for the first 11 months of recruitment) in the acute phase (≤14 days post-stroke) following clinically and/or radiologically (CT and/or MRI) confirmed diagnosis of ischemic stroke.

#### Exclusion criteria

Comorbid progressive neurological or neurodegenerative condition, major psychiatric disorder (pre-stroke history of mood disorder or stable antidepressant medication did not lead to exclusion), history of major substance abuse problems, clinically unstable, unable to give informed consent or unable to cooperate with the study protocol (e.g. due to severe aphasia, uncorrected impairment of hearing or vision). Co-recruitment with intervention studies with potential impact on mood/cognition was not allowed.

In addition, participants were asked to nominate an informant (optional).

### Recruitment

Participants were recruited from acute stroke units within NHS Greater Glasgow and Clyde to have baseline assessments within four weeks of stroke onset. The Scottish Stroke Research Network nurses approached potential participants at the ward who were enrolled between 12 January 2015 and 28 January 2016. Participants were advised the study investigated listening-based leisure activities to avoid emphasizing music as an active ingredient. Participants received no financial support for their participation in the study.

### Assessments and randomization

Baseline cognitive status and mood were assessed using standardized assessments (Table 1). The order of presentation was standardized to prioritize a core set of tests while getting participants to complete as many tests from the full assessment battery as feasible. Prior to unblinding and data analysis, we selected primary outcome measures for cognition (Delayed Story Recall) and mood (HADS) based on previous findings,^5^ clinical importance, expected sensitivity to the intervention and feasibility of administration in a full trial.

**Table 1.** Assessments

Cognitive domain	
Global Cognition	Montreal Cognitive Assessment (MoCA)^14^
Attention	Test of Everyday Attention (TEA)^15^: Map search; Elevator counting; Elevator counting with distraction; Visual Elevator; Telephone search; Telephone search with counting.
Sustained attention	Sustained Attention to Response Task (SART)^16^; TEA Lottery^15^
Verbal memory	BIRT Memory and Information Processing Battery (BMIPB)^17^ subtests: (i) Story recall (immediate and delayed); (ii) List learning
Speed of processing	BMIPB^17^ Speed of information processing
Reaction time	CANTAB^18^ 5-choice reaction time subtest
Verbal working memory	Wechsler Adult Intelligence Scale – Fourth Edition (WAIS-IV)^19^; Digit Span
Visual working memory	Wechsler Memory Scale – Fourth Edition (WMS-IV)^20^ Symbol Span
Phonemic fluency	Controlled Oral Word Association Test^21^
	**Questionnaire**
Mood	Hospital Anxiety and Depression Scale (HADS)^22^
Attentional control	Metacognitions Questionnaire short form (MCQ-30)^23^
Mindfulness	Five Facet Mindfulness Questionnaire short form (FFMQ-SF)^24^
Emotion regulation	Brain Injury Rehabilitation Trust Regulation of Emotions Questionnaire (BREQ)^25^
Functional independence	Mayo-Portland Adaptability Inventory-4 (MPAI-4)^26^ (Baseline + 6-months only)

Following completion of the baseline assessments, stratified randomization with blocking (block size 3, allocation ratio 1:1:1) was used to allocate participants to groups based on: (i) recruitment location, (ii) type of stroke (cortical vs. subcortical), and (iii) recurrence (first vs. recurrent stroke). Randomization was via an automated telephone system. Being a single blind study, participants were not blind to intervention group allocation.

### Interventions

All interventions followed a manualized eight-week program developed by the research team and delivered by an assistant psychologist on an individual basis. Participants were given an iPod Nano (7th Generation, Apple Inc.) and asked to listen to their material daily on their own for at least an hour during the intervention phase (target, 56 h over eight consecutive weeks). They were also asked to keep a written daily record of listening though which adherence to the interventions was measured. Written instructions were provided. Participants selected their preferred music/audiobooks from any genre with the therapist keeping a record of the genres given at each visit. The therapist also rated their perceived feasibility of intervention delivery at each session as ’fully feasible’, ‘partially feasible’ or ‘not feasible’.

The mindful-music participants were introduced to the concept of mindfulness at the first visit and given a recording containing a brief mindfulness exercise (Body scan) to complete daily prior to music listening (weeks 1–3). The brief (∼5 min) mindfulness exercises^8^ focused on key elements of mindfulness (e.g. paying attention to the present moment). If participants were to notice any thoughts or sensations arising either during the brief exercise or during subsequent music listening, they were to allow them to pass and to gently bring their attention back to the exercise/music. Seven more weekly visits followed during which progress was monitored, listening records collected and additional material provided as required. Adherence to the daily mindfulness exercises was measured via participants’ diary records. At the fourth visit, another brief exercise (Following the breath) was provided for use over the following three weeks (weeks 4–6). For the last two weeks, participants could choose which exercise to complete. The two music groups therefore differed by the virtue of attentional control with the mindful-music group asked to return their attention back to the music whenever their mind had wandered with no specific listening instructions given to the music only group.

At the final visit, plans for listening post-intervention were discussed and CD/recording of the mindfulness exercises given to the mindful-music group.

Post-intervention, participants were interviewed about their experience of engaging in the trial, including participant perceived feasibility and acceptability of the interventions. These findings are reported elsewhere.^8^

### Follow-up

Follow-up assessments of mood and cognition using parallel versions where available (Table 1) were completed post-intervention and at six months post-stroke by an assessor blind to group allocation. Information on medication and other interventions were also collected. Informants provided ratings of emotion regulation and functional independence at six months post-stroke.

### Treatment fidelity

All intervention sessions were audio-recorded. A random sub-sample (*n* = 18, 5%) were checked for treatment fidelity by the principal investigator (JE) who was not involved in intervention delivery or outcome assessments. The selection was done using an Excel random number generator with approximately equal numbers selected from the beginning (sessions 1–3), middle (sessions 4–5), and end (sessions 6–8) of the intervention. Adherence was rated using a three-point scale (content consistent with protocol for stage of treatment; partially consistent but evidence of deviation into other unrelated areas or treatment methods; largely inconsistent with protocol for stage of treatment).

### Statistical methods

Data were analyzed using SAS software (v9.3), following a statistical analysis plan, on an intention-to-treat basis. Missing data were not imputed. Continuous variables were summarized as mean and standard deviation (SD) or median and interquartile range (IQR), depending on distribution. Categorical variables were summarized as number and percentage (n(%)). Between-group differences were assessed using analysis of variance (ANOVA), Kruskal–Wallis, or Fisher’s tests, as appropriate. Effect sizes were calculated using linear regression models, adjusted for baseline measure and stratification factors (recruitment location/type of stroke/stroke recurrence). The study was not designed to have power to detect significant differences in outcomes between groups.

## Results

### Feasibility of recruitment

**Figure 2.**
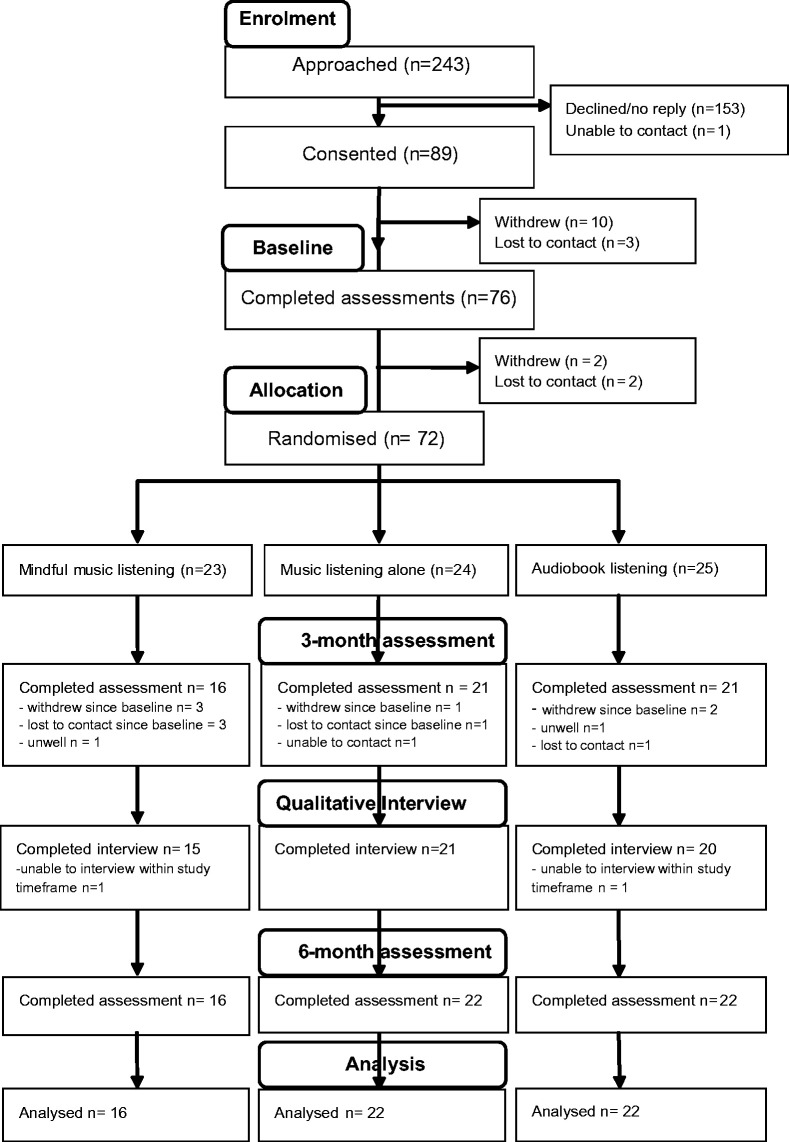
CONSORT Flowchart.

Feasibility of recruitment is shown in Figure 2.

Reasons for withdrawal prior to randomization did not appear to relate to the intervention per se but related to feeling too unwell in general (n3), feeling overwhelmed following hospital discharge (n2), deciding to focus on speech and language therapy (n1), change of mind (n2) or change in living arrangements (n2). Some gave no specific reason (n7).

Sixty-two (86.1%) of those randomized nominated an informant to complete questionnaires at the six-month follow-up. Forty-eight (77.4%) consented to receiving the questionnaires, of which 34 (73.8%) were returned by a spouse/partner (41.5%), child (18.5%), friend (6.2%), parent (4.6%), sibling (1.5%), and other relative (1.5%).

The average rate of recruitment was 1.6 participants per week (89 participants/55 weeks).

### Sample characteristics

Sample characteristics at baseline are presented in Table 2. A summary of cognitive and mood status, medication and other interventions including engagement in listening and mindfulness-based leisure activities pre-stroke is provided in the Supplementary Tables 1 to 3.

**Table 2.** Baseline participant characteristics

		Overall (*N* = 72)	Mindfulness (*N* = 23)	Music (*N* = 24)	Audiobook (*N* = 25)
Age (years)	Mean (SD)	64.0 (11.60)	65.3 (11.13)	61.1 (10.36)	65.7 (12.97)
Gender *n* (%)	Male	45 (62.5%)	13 (56.5%)	17 (70.8%)	15 (60.0%)
Education (years)	Median (IQR)	11.0 (10.0, 15.0)	12.0 (10.0, 15.0)	11.2 (10.5, 15.0)	10.0 (10.0, 13.0)
SIMD quintile	0–20%	32(44.4%)	6 (26.1%)	13 (54.2%)	13 (52.0%)
0% most deprived,	20–40%	14(19.4%)	7(30.4%)	5(20.8%)	2(8.0%)
100% least deprived	40–60%	9 (12.5%)	4 (17.4%)	2 (8.3%)	3 (12.0%)
	60–80%	10 (13.9%)	2 (8.7%)	2 (8.3%)	6 (24.0%)
	80–100%	7 (9.7%)	4(17.4%)	2 (8.3%)	1 (4.0%)
Oxford stroke classification	Cortical	46 (63.9%)	15 (65.2%)	15 (62.5%)	16 (64.0%)
	Subcortical	26 (36.1%)	8(34.8%)	9 (37.5%)	9 (36.0%)
If cortical, type	TACS	3 (6.5%)	1 (6.7%)	1 (6.7%)	1 (6.3%)
	PACS	21 (45.7%)	9 (60.0%)	5 (33.3%)	7 (43.8%)
	POCS	22 (47.8%)	5(33.3%)	9 (60.0%)	8 (50.0%)
Hemisphere	Right	27 (38.0%)	6 (27.3%)	10 (41.7%)	11 (44.0%)
	Left	40 (56.3%)	15 (68.2%)	14 (58.3%)	11 (44.0%)
	Other	4 (5.6%)	1 (4.5%)	0	3 (12.0%)
Stroke recurrence	First	59 (81.9%)	19 (82.6%)	20 (83.3%)	20 (80.0%)
	Second	12 (16.7%)	3 (13.0%)	4 (16.7%)	5 (20.0%)
	Third or more	1 (1.4%)	1 (4.3%)	0	0
Modified Rankin scale	0 (no symptoms)	5 (6.9%)	4 (17.4%)	0	1(4.0%)
	1	22 (30.7%)	7 (30.4%)	9 (37.5%)	6 (24.0%)
	2	33 (45.8%)	7 (30.4%)	14 (58.3%)	12 (48.0%)
	3	6 (8.3%)	3 (13.0%)	0	3 (12.0%)
	4 (Moderately severe disability)	6 (8.3%)	2 (8.7%)	1 (4.2%)	3 (12.0%)

IQR: interquartile range; PACS: partial anterior circulation stroke; POCS: posterior circulation stroke; SD: standard deviation; SIMD: Scottish index of multiple deprivation; TACS: total anterior circulation stroke

The median time from stroke onset to baseline assessment was 19 days (range 5–28).

### Retention during intervention

Of the 72 participants randomized, 65 (90.3%) commenced treatment. Of those who did not commence, four withdrew after randomization (mindful-music (n2), audiobooks (n2)), one was lost to contact, and two were unable to commence for other reasons. Median time from stroke to the first intervention session was 31 days (range 17–56). Seven individuals dropped out of the intervention but remained in the study. One dropped out of the mindful-music group (one session completed) due to difficulty concentrating in general, two from the music group (one after one session and one after two sessions) due to group allocation, high number of other treatments, and four from the audiobooks (no, two, three and four sessions completed) due to group allocation, low mood, and issues with visit scheduling (e.g. staying with family in another part of the country). Data from these individuals have been included in statistical analyses. Two withdrew from the study during the intervention phase due to being unwell.

### Feasibility and acceptability of interventions

Intervention feasibility was assessed via therapist ratings with 96.9% of treatment sessions rated ‘fully feasible’ by the therapist. The remaining 3.1% were rated ‘partially feasible’ due to fatigue, hand function, mood, comprehension, verbal expression, cognition or session content. The median treatment session completion was 7 for mindful-music, 6 for music, and 8 for audiobooks with 68.1% completing at least six intervention sessions and 37.5% completing all eight sessions. The majority (86.6%) of sessions took place at the participant’s home, followed by work (8.4%), telephone (2.5%), hospital (2.2%), and e-mail (0.2%). The mean first intervention session length was 75 min (SD 29) for mindful-music, 63 min (SD 17) for music, and 59 min (SD 21) for the audiobook group (*p* = 0.24). Subsequent sessions were approximately 20 min. The mean listening diary completion rate over eight weeks for those randomized was 68% (0–100%) and 80% (0–100%) for those completing the study.

Adherence to music/audiobook listening and mindfulness exercises was measured via participants’ diary records against the recommended dose (1 h × 7 days × 8 weeks = 56 h). Median listening time in the mindful-music group was 45 h (80.4% of target listening), 60 h in the music group (107% of target listening), and 49 h (87.5% of target listening) in the audiobook group which did not differ statistically (p = 0.15). The mean adherence to the mindfulness exercises in the mindful-music group was 40% (0–100%) for those randomized and 60% (0–100%) for those completing the six-month follow-up. New listening material was provided in 59% of sessions, with fiction/crime being most popular for audiobooks, pop/rock in the music group, and classical/easy listening in the mindful-music group. As described elsewhere,^8^ 94.6% of participants found appointments convenient and 73.3% reported it was feasible to listen daily for up to an hour. The music and audiobook groups were more likely to engage in daily music listening post-intervention compared to the mindful-music group (*p* = 0.012). Similarly, the audiobook group was more likely to continue audiobook listening post-intervention compared to the other two groups (*p* = 0.0052). No significant group differences (*p* = 0.31) were found in engagement in relaxation, mindfulness or meditation-based leisure activities (Supplementary Table 4).

### Retention to six-month end-point

Retention to the six-month end-point is summarized in Figure 2 and was similar across groups (*p* = 0.13). For the mindful-music group, seven were lost (four withdrew due to ill-health [prior intervention (n2), after session 4 (n1) and at qualitative interview (n1)] and three were lost to contact [prior intervention (n1) and during the intervention (n2)]). For the music group, two were lost (one withdrew due to ill-health and one was lost to contact during the intervention). For the audiobook group, three were lost (one withdrew prior intervention due to ill-health, one immediately after randomization due to unwillingness to listen to audiobooks and one was lost to contact at three-month follow-up). Withdrawals due to ill-health did not appear to be related to the interventions but to other health problems or the effects of stroke (e.g. feeling overwhelmed after returning home from hospital).

### Treatment fidelity

Treatment fidelity was judged to be high with 17/18 (94.4%) sessions being fully consistent with the study protocol.

### Effect sizes and sample size calculations for full-scale trial

The study was not designed to evaluate differences in outcomes between groups; however, intention to treat analysis was carried out to obtain an estimate of treatment effect (Cohen’s d) on outcomes at three and six months post-stroke. Cohen^27^ suggested that effect sizes are classed as small (d = 0.2), medium (d = 0.5), and large (d = 0.8). Results of this analysis are provided in the Supplementary Tables 5 and 6. With regard to the outcome measures chosen a priori for a future full-scale trial, it is noteworthy that Delayed Story Recall produced one of the largest effect sizes (standardized mean difference) at six months, favoring the music (d = 0.42 [−0.07, 0.92]) and the mindful-music (d = 0.44 [−0.11, 1.00]) groups (small-medium effect), compared with audiobooks. Similar findings were apparent for Immediate Story Recall favoring the music (d = 0.58 [0.06, 1.11]) and mindful-music (d = 0.51 [−0.07, 1.09]) groups (medium-large effect) over audiobooks; and for the measure of attentional switching (visual elevator accuracy score), which also favored the music (d = 0.67 [0.12, 1.22]) and mindful-music (d = 0.77 [0.16, 1.38]) groups (medium-large effect) over audiobooks. The mean HADS mood scores were within the normal range across groups at all time points. Many of the other measures of cognition and the self-report questionnaires showed very little difference between groups.

Measures of variance in change scores were used to estimate sample size for a full-scale trial. Based on Delayed Story Recall at six months, a full-scale three-arm trial would require the randomization of 306 participants to detect a clinically substantial difference in improvement (z-score difference = 0.66, *p* = 0.017 [Bonferroni-correction], two-tailed, 80% power, with 16.7% attrition). This would also provide 91% power to detect a modest (2-point) difference in change in depression and 84% power to detect a 2-point difference in change in anxiety measured using the HADS.

## Discussion

Recruitment to MELLO was feasible, though slower than anticipated. The interventions were feasible to deliver and acceptable to participants with high treatment fidelity. Listening adherence was found to be excellent with the music listening group exceeding their listening target and the other two arms achieving at least 80% of their target listening time with the both music only and audiobook groups found to be significantly more likely to continue listening to their allocated material during the three month follow-up phase compared to the mindful music listening group. All treatment arms had attrition rates equal to or better than what might be typically expected for psychological therapies post-stroke.^28^ Our recruitment and adherence results are also aligned with previous studies of post-stroke music listening.^5,29^ The study was not powered as an efficacy trial but treatment effect sizes for both music listening groups compared to audiobook listening at six-months on measures identified a-priori as key outcomes (verbal memory and attentional switching), were consistent with previous research.^5^ One must be cautious, however, in interpreting effect sizes, particularly since some measures had missing data at one or more time points. We have previously reported positive self-reported cognitive impacts in the domains of memory and attention based on participants’ qualitative experiences of the interventions.^8^ Both music groups but not the audiobook group reported memory reminiscence, while the mindful music group referred to being better able to refocus their mind following mind wandering consistent with the idea of improved attentional control or attentional switching.

Our sample had low levels of depression and anxiety throughout the study. It is possible that participants with, or vulnerable to, mood disorder chose not to participate (sampling bias), or that all groups benefitted from a listening-based activity and weekly contact. The latter is partially supported by feedback from post-intervention interviews^8^ suggested that participants in the music listening groups more frequently referred to listening being enjoyable and uplifting, and promoting memory reminiscence compared to audiobooks. But there were differences between the music groups, with the mindful-music group more frequently referring to listening aiding relaxation, attentional control, and emotion regulation.

A strength of our study was inclusion of a clinically and demographically diverse population compared to the previous music listening study in stroke.^5^ As with other acute stroke, interventional studies numbers with moderate to severe stroke were low. In contrast to Särkämö et al.,^5^ we did not use a qualified music therapist to deliver the interventions, given the limited availability of this expertise within the UK National Health Service. Similar findings between the studies suggest this may not be critical.

We recognize limitations in our study but can use these to improve design of a future trial. Given the nature of cognitive, physical, and psychological difficulties in our participants, it was not possible to administer all tests to every participant at each time point. We therefore prioritized a core set of tests, albeit attempted to administer as many of the planned assessments as was feasible without over-burdening our participants. Future trials should limit the number of tests administered and the finding that medium-large effect sizes for measures of verbal memory (Story Recall) and attentional switching were consistent with previous research suggests these would be appropriate for future studies.

Other limitations were the use of self-reported listening diaries. Although the diaries were completed daily and collected on a weekly basis, estimation of listening duration is subject to bias. A future trial would benefit from using an objective measure of listening duration. Future trials could also explore options for using technology or existing services to support listening and examine whether using music in different ways (for relaxation, to improve concentration) affects outcomes or improves engagement in other therapies (e.g. physiotherapy). Research should also investigate potential mechanisms of action. One possibility may be improved attentional control, but there are other potential psychological and neurobiological mechanisms such as emotion regulation, reward and stress reduction, which may mediate the cognitive effects of music^30^ and mindfulness-based interventions.^31^

In summary, we found that it was feasible and acceptable to incorporate brief mindfulness training into a music listening intervention in an RCT context post-stroke. Although effect sizes for key cognitive (memory and attention) variables were consistent with previous research in favoring the music listening interventions over the audiobook control, a full scale definitive RCT is needed before treatment recommendations could be made. Recruitment and retention data, in combination with power analysis, suggest that progression to a full-scale trial seems feasible and justified.

## Supplemental Material

Supplemental material for Measuring the effects of listening for leisure on outcome after stroke (MELLO): A pilot randomized controlled trial of mindful music listening Supplemental Material for Measuring the effects of listening for leisure on outcome after stroke (MELLO): A pilot randomized controlled trial of mindful music listening by Satu Baylan, Caroline Haig, Maxine MacDonald, Ciara Stiles, Jake Easto, Meigan Thomson, Breda Cullen, Terence J Quinn, David Stott, Stewart W Mercer, Niall M Broomfield, Heather Murray and Jonathan J Evans in International Journal of Stroke
