# Supplementary material for: Measuring the effects of listening for leisure on outcome after stroke (MELLO): A pilot randomized controlled trial of mindful music listening
Source: Int J Stroke. 2019 Apr 2;15(2):149–58. doi: 10.1177/1747493019841250 (PMC7045280; doi:10.1177/1747493019841250)
Supplement: Supplemental material for Measuring the effects of listening for leisure on outcome after stroke (MELLO): A pilot randomized controlled trial of mindful music listening [file Supplemental_Material.pdf]

SUPPLEMENTARY MATERIAL

Baylan et al., Measuring the Effects of Listening for Leisure on Outcome after stroke (MELLO): a pilot randomised controlled trial of mindful music listening. International Journal of Stroke, 2019.

Supplementary Table 1: Baseline cognitive and mood characteristics

		**Overall (N=72)**	**Mindfulness (N=23)**	**Music (N=24)**	**Audiobook (N=25)**
MoCA: total (/30)	Mean (SD)	23.0 (4.13)	22.7 (4.76)	23.9 (2.91)	22.5 (4.53)
Symbol span: scaled	Median (IQR)	7.0 (6.0,10.0)	8.0 (6.0,11.0)	7.0 (6.0,9.5)	7.0 (6.0,10.0)
Map search 1-min	Median (IQR)	20.5 (15.0,28.5)	20.0 (12.0,30.0)	20.5 (16.5,27.0)	21.0 (14.0,26.0)
Map search 2-min	Median (IQR)	37.5 (27.0,54.0)	37.0 (25.0,60.0)	38.5 (29.0,50.0)	35.0 (23.0,51.0)
Elevator counting classification	Normal	50 (69.4%)	14 (60.9%)	18 (75.0%)	18 (72.0%)
	Possibly abnormal	15 (20.8%)	6 (26.1%)	6 (25.0%)	3 (12.0%)
	Abnormal	7 (9.7%)	3 (13.0%)	0	4 (16.0%)
Elevator counting with distraction (/10)	Median (IQR)	6.0 (3.0,9.0)	5.0 (1.0,8.0)	7.0 (4.0,9.5)	7.0 (4.0,9.0)
Elevator visual accuracy (/10)	Median (IQR)	6.0 (3.5,9.0)	6.0 (3.0,9.0)	6.5 (5.0,8.0)	7.0 (2.0,9.0)
Telephone number search alone	N (missing)	55 (17)	17 (6)	21 (3)	17 (8)
	Median (IQR)	4.7 (3.7,6.2)	4.4 (3.8,7.0)	4.7 (4.2,5.0)	4.4 (3.6,6.5)
Telephone number search while dual tasking	N (missing)	55 (17)	17 (6)	21 (3)	17 (8)
	Median (IQR)	2.8 (0.6,9.0)	2.8 (1.0,11.9)	3.0 (1.0,6.1)	1.7 (0.2,5.2)
Lottery task (/10)	N (missing)	35 (37)	10 (13)	12 (12)	13 (12)
	Median (IQR)	9.0 (8.0,9.0)	8.5 (7.0,9.0)	9.0 (8.0,10.0)	8.0 (8.0,9.0)
BMIPB immediate recall (z-score)	N (missing)	71 (1)	23 (0)	23 (1)	25 (0)
	Mean (SD)	-0.08 (1.10)	-0.12 (1.03)	-0.20 (1.09)	0.08 (1.21)
BMIPB delayed recall (z-score)	N (missing)	71 (1)	23 (0)	23 (1)	25 (0)
	Mean (SD)	-0.08 (1.05)	-0.05 (0.99)	-0.16 (1.13)	-0.04 (1.06)
BMIPB List learning A1-A5 (z-score)	N (missing)	42 (30)	14 (9)	14 (10)	14 (11)
	Mean (SD)	-0.79 (0.99)	-0.87 (0.80)	-0.65 (1.27)	-0.86 (0.90)
BMIPB List learning A6 (z-score)	N (missing)	42 (30)	14 (9)	14 (10)	14 (11)
	Mean (SD)	-0.53 (1.07)	-0.67 (0.81)	-0.25 (1.34)	-0.67 (1.00)
Speed of information processing (z-score)	N (missing)	41 (31)	13 (10)	14 (10)	14 (11)
	Mean (SD)	-0.69 (0.87)	-0.98 (0.86)	-0.80 (0.66)	-0.32 (1.00)
Digit span overall scaled Score	N (missing)	40 (32)	13 (10)	14 (10)	13 (12)
	Mean (SD)	9.4 (2.46)	10.0 (2.86)	9.9 (2.34)	8.3 (1.89)

Baylan et al., Measuring the Effects of Listening for Leisure on Outcome after stroke (MELLO): a pilot randomised controlled trial of mindful music listening. International Journal of Stroke, 2019.

		**Overall (N=72)**	**Mindfulness (N=23)**	**Music (N=24)**	**Audiobook (N=25)**
Verbal fluency (T-score)	N (missing)	41 (31)	14 (9)	14 (10)	13 (12)
	Mean (SD)	43.5 (11.70)	39.3 (12.08)	49.5 (10.80)	41.6(10.27)
SART omissions	N (missing)	48 (24)	15 (8)	18 (6)	15 (10)
	Median (IQR)	22.0 (17.0,29.5)	22.0 (19.0,43.0)	22.5 (17.0,25.0)	24.0 (15.0,28.0)
SART commissions	N (missing)	48 (24)	15 (8)	18 (6)	15 (10)
	Median (IQR)	9.0 (5.0,13.0)	6.00 (4.00,13.00)	7.50 (3.00,13.00)	10.00 (8.00,14.00)
SART mean corrected reaction time (RT)	N (missing)	48 (24)	15 (8)	18 (6)	15 (10)
	Mean (SD)	451.6 (76.74)	469.8 (85.96)	459.9 (77.36)	423.3 (61.75)
CANTAB 5-choice RT raw score	N (missing)	39 (33)	13 (10)	12 (12)	14 (11)
	Median (IQR)	386.1 (358.1,477.4)	416.0 (354.0,477.4)	380.4 (368.3,421.3)	381.8 (353.1,486.5)
HADS depression	N (missing)	65 (7)	19 (4)	24 (0)	22 (3)
	Median (IQR)	6.0 (2.0, 8.0)	5.0 (2.0, 7.0)	6.5 (2.0, 11.0)	6.5 (2.0, 8.0)
HADS anxiety	N (missing)	66 (6)	20 (3)	24 (0)	22 (3)
	Median (IQR)	7.0 (3.0,11.0)	7.0 (6.0,9.5)	5.0 (1.5,11.5)	7.0 (3.00,12.0)

Data are raw scores unless stated otherwise.

BMIPB, BIRT Memory and Information Processing Battery; HADS, Hospital Anxiety and Depression Scale; IQR, inter quartile range; MoCA, Montreal Cognitive Assessment; Min, minute(s), SART, Sustained Attention Response Test; SD, standard deviation

SUPPLEMENTARY MATERIAL

Baylan et al., Measuring the Effects of Listening for Leisure on Outcome after stroke (MELLO): a pilot randomised controlled trial of mindful music listening. International Journal of Stroke, 2019.

Supplementary Table 2:

a. Medication and interventions at baseline

		Overall (N=72)	Mindfulness (N=23)	Music (N=24)	Audiobook (N=25)
Medication	Unknown	2 (2.8%)	1 (4.3%)	0	1 (4%)
antidepressant	Yes	14 (20.0%)	7 (31.8%)	3 (12.5%)	4 (16.7%)
antipsychotic	Yes	1 (1.4%)	1 (4.5%)	0	0
mood stabiliser	Yes	2 (2.9%)	1 (4.5%)	1 (4.2%)	0
pain medication	Yes	33 (47.1%)	8 (36.4%)	15 (62.5%)	10 (41.7%)
Receiving any	Yes	24 (33.3%)	5 (21.7%)	9 (37.5%)	10 (40.0%)
Interventions	No	45 (62.5%)	17 (73.9%)	14 (58.3%)	14 (56.0%)
	Not returned	3 (4.2%)	1 (4.3%)	1 (4.2%)	1 (4.0%)
If yes, Physiotherapy	Yes	15 (62.5%)	5 (100.0%)	5 (55.6%)	5 (50.0%)
OT	Yes	12 (50.0%)	3 (60.0%)	5 (55.6%)	4 (40.0%)
SALT	Yes	2 (8.3%)	0	0	2 (20.0%)
Psychology	Yes	0	0	0	0
Psychiatry	Yes	1 (4.2%)	0	1 (11.1%)	0
Other	Yes	4 (16.7%)	0	1 (11.1%)	3 (30.0%)

OT, Occupational Therapy; SALT, Speech and Language Therapy;

b. Medication and interventions at 3-months post-stroke

		Overall (N=58)	Mindfulness (N=16)	Music (N=21)	Audiobook (N=21)
Medication					
antidepressant	Yes	10 (17.0%)	2 (12.5%)	3 (13.6%)	5 (23.8%)
antipsychotic	Yes	1 (1.7%)	0	0	1 (4.8%)
mood stabiliser	Yes	2 (3.4%)	1 (6.3%)	1 (4.6%)	0
pain medication	Yes	27 (45.8%)	6 (37.5%)	11 (50.0%)	10 (47.6%)
Receiving any	Yes	19 (29.2%)	6 (31.6%)	7 (30.4%)	6 (26.1%)
Interventions	No	38 (58.5%)	10 (52.6%)	14 (60.9%)	14 (60.9%)
	Not returned	8 (12.3%)	3 (15.8%)	2 (15.8%)	3 (13.05%)
If yes, Physiotherapy	Yes	10 (52.6%)	3 (50.0%)	3 (43.9%)	5 (50.0%)
OT	Yes	8 (42.1%)	2 (33.3%)	4 (57.1%)	2 (33.3%)
SALT	Yes	4 (21.0%)	2 (33.3%)	1 (14.9%)	1 (16.7%)
Psychology	Yes	3 (15.8%)	1 (16.7%)	2 (28.6%)	0
Psychiatry	Yes	2 (10.5%)	0	1 (14.3%)	1 (16.7%)
Other	Yes	4 (21.0%)	1 (16.7%)	2 (28.6%)	1 (16.7%)

OT, Occupational Therapy; SALT, Speech and Language Therapy

Baylan et al., Measuring the Effects of Listening for Leisure on Outcome after stroke (MELLO): a pilot randomised controlled trial of mindful music listening. International Journal of Stroke, 2019.

c. Medication and interventions at 6-months post-stroke

		**Overall** (N=60)	**Mindfulness** (N=16)	**Music** (N=22)	**Audiobook** (N=22)
Medication					
antidepressant	Yes	14 (24.1%)	3 (18.8%)	6 (28.6%)	5 (23.8%)
antipsychotic	Yes	0	0	0	0
mood stabiliser	Yes	2 (3.5%)	1 (6.3%)	1 (4.7%)	0
pain medication	Yes	22 (37.9%)	4 (25.0%)	9 (42.9%)	9 (42.9%)
Receiving any	Yes	16 (24.6%)	3 (15.8%)	4 (17.4%)	9 (39.1%)
Interventions	No	43 (66.2%)	13 (68.4%)	17 (73.9%)	13 (56.5%)
	Not returned	6 (9.2%)	3 (15.8%)	2 (8.7%)	1 (4.4%)
If yes, Physiotherapy	Yes	8 (50.0%)	1 (33.3%)	2 (50.0%)	5 (55.6%)
OT	Yes	3 (18.8%)	1 (33.3%)	1 (25.0%)	1 (11.1%)
SALT	Yes	1 (6.3%)	0	1 (25.0%)	0
Psychology	Yes	6 (37.5%)	2 (66.7%)	1 (25.0%)	3 (33.3%)
Psychiatry	Yes	2 (12.5%)	0	0	2 (22.2%)
Other	Yes	2 (12.5%)	1 (33.3%)	1 (25.0%)	0

OT, Occupational Therapy; SALT, Speech and Language Therapy

Baylan et al., Measuring the Effects of Listening for Leisure on Outcome after stroke (MELLO): a pilot randomised controlled trial of mindful music listening. International Journal of Stroke, 2019.

Supplementary Table 3. Engagement in music and mindfulness based leisure activities in the year before stroke

**Activity**	**Frequency**	**Overall** (n = 72)	**Mindful music** (n =23)	**Music** (n = 24)	**Audiobook** (n = 25)
Music listening	Never<br>Some<br>missing	5 (7.0%)<br>66 (92.6%)<br>1	1 (4.6%)<br>21 (95.4%)<br>1	3 (12.5%)<br>21 (87.5%)<br>0	1 (4.0%)<br>24 (96.0%)<br>0
Singing or playing an instrument	Never<br>Some	61 (84.7%)<br>11 (15.3%)	17 (73.9%)<br>6 (26.1%)	23 (95.8%)<br>1 (4.2%)	21 (84.0%)<br>4 (16.0%)
Relaxation, mindfulness or meditation	Never<br>Some	60 (83.3%)<br>12 (16.7%)	20 (87.0%)<br>3 (13.0%)	19 (79.2%)<br>5 (20.8%)	21 (84.0%)<br>4 (16.0%)

Baylan et al., Measuring the Effects of Listening for Leisure on Outcome after stroke (MELLO): a pilot randomised controlled trial of mindful music listening. International Journal of Stroke, 2019.

Supplementary Table 4: Self-reported engagement in listening and mindfulness based leisure activities between 3 and 6 months post-stroke

**Activity**	**Categories**	**Mindful music** (n=15)	**Music** (n=21)	**Audiobook** (n=21)	**P-value Fisher**
Music listening	Never	0	2 (9.5%)	0	0.012
	Less than once per month	0	0	2 (9.5%)	
	1-3 times per month	0	0	2 (9.5%)	
	1-2 times per week	3 (20.0%)	1 (4.8%)	2 (9.5%)	
	3-4 times per week	7 (46.7%)	5 (23.8%)	1 (4.8%)	
	Daily/almost daily	5 (33.3%)	13 (61.9%)	14 (66.7%)	
Audiobook listening	Never	14 (93.3%)	20 (95.2%)	12 (57.1%)	0.0052
	Less than once per month	1 (6.7%)	1 (4.8%)	0	
	1-3 times per month	0	0	2 (9.5%)	
	1-2 times per week	0	0	2 (9.5%)	
	3-4 times per week	0	0	1 (4.8%)	
	Daily/almost daily	0	0	4 (19.1%)	
Relaxation, mindfulness or meditation	Never	5 (33.3%)	15 (71.4%)	15 (68.2%)	0.31
	Less than once per month	3 (20.0%)	1 (4.8%)	4 (18.2%)	
	1-3 times per month	2 (13.3%)	2 (9.5%)	1 (4.6%)	
	1-2 times per week	1 (6.7%)	0	0	
	3-4 times per week	3 (20.0%)	2 (9.5%)	1 (4.6%)	
	Daily/almost daily	1 (6.7%)	1 (4.8%)	1 (4.6%)	

Baylan et al., Measuring the Effects of Listening for Leisure on Outcome after stroke (MELLO): a pilot randomised controlled trial of mindful music listening. International Journal of Stroke, 2019.

Supplementary Table 5: Three month assessment adjusted mean differences and effect sizes (Cohen's d) for differences in change scores for each of the measures, with comparisons between Mindful music listening and audiobook, and between music listening and audiobook. Positive effect sizes favour the Mindful music/Music groups over the Audiobook group.

Assessment (n)	Group (ref audiobook)	Adjusted mean difference (95% CI)	Effect size d (95% CI)
MoCA total score (n57)	Mindfulness	0.63 (-0.90, 2.17)	0.20 (-0.28, 0.67)
	Music	1.81 (0.38, 3.25)	0.56 (0.12, 1.01)
BMIPB immediate story recall Recall (age norm z-score) (n55)	Mindfulness	0.71 (-0.04, 1.47)	0.60 (-0.03, 1.23)
	Music	0.75 (0.06, 1.45)	0.63 (0.05, 1.22)
BMIPB delayed story recall (age norm z-score) (n55)	Mindfulness	0.60 (-0.14, 1.34)	0.48 (-0.11, 1.06)
	Music	0.61 (-0.07, 1.29)	0.48 (-0.05, 1.02)
BMIPB List learning A1-A5 (age norm z-score) (n34)	Mindfulness	0.76 (-0.03, 1.55)	0.74 (-0.03, 1.51)
	Music	0.49 (-0.23, 1.21)	0.48 (-0.23, 1.18)
BMIPB List learning A6 (age norm z- score) (n34)	Mindfulness	0.57 (-0.32, 1.46)	0.58 (-0.33, 1.49)
	Music	0.59 (-0.22, 1.40)	0.60 (-0.23, 1.42)
BMIPB Speed of information information processing (z score) (n33)	Mindfulness	0.09 (-0.43, 0.60)	0.11 (-0.54, 0.76)
	Music	0.16 (-0.29, 0.62)	0.21 (-0.36, 0.78)
TEA elevator counting with distraction (n56)	Mindfulness	0.80 (-0.62, 2.23)	0.27 (-0.21, 0.75)
	Music	-0.01 (-1.33, 1.31)	-0.00 (-0.45, 0.44)
TEA visual elevator accuracy score (n56)	Mindfulness	0.45 (-1.01, 1.92)	0.18 (-0.40, 0.75)
	Music	0.28 (-1.07, 1.63)	0.11 (-0.42, 0.64)
TEA visual elevator timing score (n54)	Mindfulness	-0.70 (-2.72, 1.31)	0.23 (-0.44, 0.91)
	Music	-0.79 (-2.70, 1.13)	0.26 (-0.37, 0.90)
TEA 1 minute map search (n56)	Mindfulness	0.82 (-2.49, 4.14)	0.09 (-0.27, 0.45)
	Music	0.06 (-2.99, 3.11)	0.01 (-0.33, 0.34)
TEA 2 minute map search (n56)	Mindfulness	-2.88 (-7.91, 2.15)	-0.18 (-0.51, 0.14)
	Music	-1.58 (-6.10, 2.94)	-0.10 (-0.39, 0.19)
TEA Telephone number search search (alone) (n47)	Mindfulness	0.14 (-0.57, 0.85)	-0.07 (-0.45, 0.30)
	Music	-0.13 (-0.79, 0.53)	0.07 (-0.28, 0.42)
TEA Telephone number search search (dual task) (n47)	Mindfulness	-0.61 (-5.71, 4.80)	0.10 (-0.72, 0.91)
	Music	-0.25 (-4.98, 4.48)	0.04 (-0.72, 0.80)
TEA Lottery task (n29)	Mindfulness	0.55 (-0.48, 1.58)	0.47 (-0.42, 1.37)
	Music	0.02 (-0.95, 0.99)	0.02 (-0.82, 0.86)
HADS Anxiety (n56)	Mindfulness	0.69 (-1.47, 2.84)	-0.16 (-0.66, 0.34)
	Music	1.35 (-0.64, 3.34)	-0.32 (-0.78, 0.15)
HADS depression (n56)	Mindfulness	0.70 (-1.65, 3.04)	-0.17 (-0.72, 0.39)
	Music	-0.14 (-2.29, 2.01)	0.03 (-0.48, 0.54)
Symbol span (scaled) (n56)	Mindfulness	0.22 (-1.24, 1.68)	0.08 (-0.45, 0.60)
	Music	0.30 (-0.99, 1.58)	0.11 (-0.36, 0.57)
Digit span forward (scaled) (n31)	Mindfulness	0.33 (-1.45, 2.10)	0.14 (-0.61, 0.89)
	Music	-0.12 (-1.67, 1.43)	-0.05 (-0.71, 0.60)

Digit span backward (scaled) (n31)	Mindfulness	1.00 (-0.94, 2.95)	0.44 (-0.42, 1.30)
	Music	0.68 (-0.98, 2.34)	0.30 (-0.44, 1.04)
Digit span seq. (scaled) (n31)	Mindfulness	0.61 (-1.57, 2.79)	0.33 (-0.84, 1.50)
	Music	-0.08 (-1.99, 1.83)	-0.04 (-1.07, 0.98)
Digit span overall (scaled) (n31)	Mindfulness	0.62 (-1.15, 2.39)	0.30 (-0.56, 1.16)
	Music	-0.05 (-1.54, 1.45)	-0.02 (-0.75, 0.70)
Verbal fluency (T-score) (n32)	Mindfulness	0.38 (-8.57, 9.32)	0.03 (-0.76, 0.82)
	Music	0.20 (-7.94, 8.34)	0.02 (-0.70, 0.74)
SART errors of omission (n40)	Mindfulness	5.25 (0.91, 9.58)	-0.52 (-0.95, -0.09)
	Music	-0.44 (-4.35, 3.46)	0.04 (-0.34, 0.43)
SART errors of commission (n40)	Mindfulness	0.97 (-2.27, 4.21)	-0.18 (-0.79, 0.42)
	Music	2.55 (-0.34, 5.44)	-0.48 (-1.02, 0.06)
SART mean RT (n40)	Mindfulness	45.88 (-7.33, 99.10)	-0.57 (-1.23, 0.09)
	Music	3.40 (-44.88, 51.68)	-0.04 (-0.64, 0.56)
CANTAB 5-choice RT (n30)	Mindfulness	-29.9 (-74.2, 14.5)	0.39 (-0.19, 0.98)
	Music	-32.6 (-73.7, 8.4)	0.43 (-0.11, 0.97)
FFMQ-SF NR subscale (n52)	Mindfulness	-0.36 (-3.34, 2.62)	-0.08 (-0.77, 0.60)
	Music	0.91 (-1.88, 3.70)	0.21 (-0.43, 0.85)
FFMQ-SF OB subscale (n52)	Mindfulness	1.60 (-0.61, 3.82)	0.38 (-0.15, 0.91)
	Music	0.61 (-1.57, 2.78)	0.15 (-0.38, 0.67)
FFMQ-SF AA subscale (n52)	Mindfulness	0.09 (-2.64, 2.82)	0.02 (-0.60, 0.64)
	Music	0.07 (-2.50, 2.63)	0.01 (-0.57, 0.60)
FFMQ-SF DS subscale (n52)	Mindfulness	0.89 (-1.22, 2.99)	0.22 (-0.30, 0.73)
	Music	-1.34 (-3.33, 0.65)	-0.33 (-0.81, 0.16)
FFMQ-SF NJ subscale (n52)	Mindfulness	0.55 (-2.54, 3.65)	0.11 (-0.52, 0.74)
	Music	-0.73 (-3.63, 2.18)	-0.15 (-0.74, 0.44)
MCQ-30 total (n50)	Mindfulness	5.22 (-2.28, 12.72)	-0.34 (-0.83, 0.15)
	Music	5.61 (-1.57, 12.79)	-0.36 (-0.83, 0.10)
BREQ-Self total (n48)	Mindfulness	0.39 (-5.52, 6.30)	-0.33 (-0.50, 0.44)
	Music	1.43 (-4.33, 7.20)	-0.11 (-0.57, 0.34)

The model was adjusted for baseline measure, recruitment location, type of stroke (cortical vs subcortical) and recurrence (first vs recurrent stroke)

BMIPB, BIRT Memory and Information Processing Battery; BREQ, Brain Injury Rehabilitation Trust Regulation of Emotions Questionnaire; FFMQ-SF, Five Faced Mindfulness Questionnaire Short Form; HADS, Hospital Anxiety and Depression Scale; MCQ-30, Metacognitions Questionnaire short form; MoCA, Montreal Cognitive Assessment; Min, minute(s), MPAI-4, Mayo-Portland Adaptability Inventory-4; SART, Sustained Attention Response Test; TEA, Test of every day attention

Baylan et al., Measuring the Effects of Listening for Leisure on Outcome after stroke (MELLO): a pilot randomised controlled trial of mindful music listening. International Journal of Stroke, 2019.

Supplementary Table 6: Six month assessment adjusted mean differences and effect sizes (Cohen's d) for differences in change scores for each of the measures, with comparisons between Mindful music listening and audiobook, and between music listening and audiobook. Positive effect sizes favour the Mindful music/Music groups over the Audiobook group.

Assessment (n)	Group (ref audiobook)	Adjusted mean difference (95% CI)	Effect size d (95% CI)
MoCA total score (n59)	Mindfulness	0.57 (-1.24, 2.38)	0.15 (-0.34, 0.65)
	Music	0.78 (-0.84, 2.40)	0.21 (-0.23, 0.65)
BMIPB immediate story recall (age norm z-score) (n56)	Mindfulness	0.75 (-0.11, 1.61)	0.51 (-0.07, 1.09)
	Music	0.86 (0.08, 1.64)	0.58 (0.06, 1.11)
BIRT delayed story recall (age norm z-score) (n56)	Mindfulness	0.73 (-0.19, 1.64)	0.44 (-0.11, 1.00)
	Music	0.70 (-0.11, 1.50)	0.42 (-0.07, 0.92)
BMIPB List learning A1-A5 (age norm z-score) (n34)	Mindfulness	0.32 (-0.37, 1.01)	0.31 (-0.36, 0.97)
	Music	0.00 (-0.66, 0.67)	0.00 (-0.64, 0.64)
BMIPB List learning A6 (age norm z-score) (n34)	Mindfulness	0.23 (-0.54, 0.99)	0.25 (-0.59, 1.09)
	Music	0.28 (-0.47, 1.03)	0.31 (-0.51, 1.13)
BMIPB Speed of score) information processing (age norm z-score) (n33)	Mindfulness	0.16 (-0.47, 0.80)	0.17 (-0.50, 0.85)
	Music	0.67 (0.08, 1.27)	0.71 (0.08, 1.34)
TEA elevator counting with distraction (n56)	Mindfulness	1.32 (-0.56, 3.21)	0.40 (-0.17, 0.97)
	Music	0.66 (-1.03, 2.35)	0.20 (-0.31, 0.71)
TEA visual elevator accuracy score (n55)	Mindfulness	1.81 (0.38, 3.25)	0.77 (0.16, 1.38)
	Music	1.58 (0.28, 2.87)	0.67 (0.12, 1.22)
TEA visual elevator timing (n52)	Mindfulness	0.82 (-0.15, 1.79)	-0.47 (-1.04, 0.09)
	Music	-0.06 (-0.96, 0.84)	0.04 (-0.49, 0.56)
TEA 1 minute map search (n58)	Mindfulness	4.47 (0.03, 8.91)	0.39 (0.00, 0.77)
	Music	3.43 (-0.47, 7.34)	0.30 (-0.04, 0.63)
TEA 2 minute map search (n58)	Mindfulness	5.24 (-1.16, 11.64)	0.31 (-0.07, 0.69)
	Music	3.22 (-2.30, 8.74)	0.19 (-0.14, 0.52)
TEA Telephone number search alone (n45)	Mindfulness	-0.17 (-0.91, 0.56)	0.09 (-0.29, 0.48)
	Music	-0.37 (-1.04, 0.31)	0.19 (-0.16, 0.55)
TEA Telephone number search (dual task) (n45)	Mindfulness	0.33 (-3.16, 3.83)	-0.09 (-0.99, 0.82)
	Music	2.63 (-0.59, 5.85)	-0.68 (-1.51, 0.15)
TEA Lottery task (n26)	Mindfulness	0.12 (-0.87, 1.04)	0.09 (-0.69, 0.88)
	Music	0.36 (-0.62, 1.35)	0.29 (-0.50, 1.08)
HADS Anxiety (n56)	Mindfulness	2.00 (-0.28, 4.28)	-0.47 (-1.01, 0.06)
	Music	1.83 (-0.21, 3.87)	-0.43 (-0.91, 0.05)
HADS depression (n57)	Mindfulness	1.02 (-1.36, 3.40)	-0.23 (-0.76, 0.31)
	Music	0.55 (-1.61, 2.72)	-0.12 (-0.61, 0.36)
Symbol span (scaled) (n58)	Mindfulness	1.04 (-0.52, 2.59)	0.36 (-0.18, 0.90)
	Music	0.72 (-0.61, 2.05)	0.25 (-0.21, 0.71)
Digit span forward (scaled) (n31)	Mindfulness	1.09 (-0.74, 2.92)	0.49 (-0.33, 1.31)
	Music	-0.12 (-1.80, 1.56)	-0.06 (-0.81, 0.70)
Digit span backward (scaled) (n31)	Mindfulness	0.52 (-1.51, 2.55)	0.18 (-0.52, 0.87)
	Music	1.32 (-0.48, 3.12)	0.45 (-0.16, 1.07)

Digit span seq. (scaled) (n31)	Mindfulness	0.85 (-1.69, 3.40)	0.26 (-0.51, 1.02)
	Music	0.56 (-1.77, 2.89)	0.17 (-0.53, 0.87)
Digit span overall (scaled) (n31)	Mindfulness	0.41 (-1.44, 2.26)	0.15 (-0.53, 0.82)
	Music	0.08 (-1.53, 1.69)	0.03 (-0.56, 0.62)
Verbal fluency (T-score) (n31)	Mindfulness	2.46 (-7.73, 12.65)	0.20 (-0.62, 1.01)
	Music	7.32 (-2.68, 17.32)	0.58 (-0.21, 1.38)
SART errors of omission (n39)	Mindfulness	3.71 (-3.63, 11.06)	-0.31 (-0.93, 0.31)
	Music	-2.16 (-8.72, 4.40)	0.18 (-0.37, 0.74)
SART errors of commission (n39)	Mindfulness	-0.62 (-4.13, 2.88)	0.13 (-0.60, 0.85)
	Music	0.34 (-2.76, 3.43)	-0.07 (-0.71, 0.57)
SART mean RT (n39)	Mindfulness	30.7 (-26.0, 87.5)	-0.40 (-1.13, 0.34)
	Music	-4.2 (-54.8, 46.4)	0.05 (-0.60, 0.71)
CANTAB 5-choice RT (n28)	Mindfulness	10.6 (-31.1, 52.4)	-0.13 (-0.65, 0.38)
	Music	34.8 (-7.6, 77.3)	-0.43 (-0.95, 0.09)
FFMQ-SF NR subscale (n52)	Mindfulness	-1.95 (-4.32, 0.34)	-0.57 (-1.23, 0.10)
	Music	1.55 (-0.60, 3.70)	0.45 (-0.17, 1.08)
FFMQ-SF OB subscale (n53)	Mindfulness	1.14 (-0.72, 2.99)	0.26 (-0.16, 0.68)
	Music	0.71 (-1.12, 2.54)	0.16 (-0.26, 0.58)
FFMQ-SF AA subscale (n52)	Mindfulness	-0.28 (-3.10, 2.55)	-0.06 (-0.66, 0.55)
	Music	0.13 (-2.52, 2.78)	0.03 (-0.54, 0.60)
FFMQ-SF DS subscale (n53)	Mindfulness	0.30 (-1.94, 2.53)	0.08 (-0.52, 0.67)
	Music	-0.05 (-2.10, 2.00)	-0.01 (-0.56, 0.53)
FFMQ-SF NJ subscale (n53)	Mindfulness	1.38 (-1.28, 4.05)	0.30 (-0.28, 0.87)
	Music	-0.13 (-2.63, 2.37)	-0.03 (-0.57, 0.51)
MCQ-30 total (n49)	Mindfulness	1.95 (-7.57, 11.49)	-0.11 (-0.68, 0.45)
	Music	0.46 (-8.83, 9.75)	-0.03 (-0.57, 0.52)
BREQ-Self total (n48)	Mindfulness	6.20 (-1.71, 14.10)	-0.42 (-0.96, 0.12)
	Music	2.39 (-5.46, 10.24)	-0.16 (-0.70, 0.37)
MPAI-4 (self) total (n36)	Mindfulness	1.06 (-7.16, 9.29)	-0.10 (-0.85, 0.66)
	Music	-2.41 (-10.45, 5.63)	0.22 (-0.52, 0.96)
MPAI-4 (self) adjustment (n38)	Mindfulness	1.99 (-6.22, 10.19)	-0.17 (-0.89, 0.54)
	Music	-4.65 (-12.49, 3.20)	0.41 (-0.28, 1.09)
MPAI-4 (self) abilities (n37)	Mindfulness	2.93 (-5.57, 11.43)	-0.26 (-1.02, 0.50)
	Music	5.27 (-2.88, 13.42)	-0.47 (-1.20, 0.26)
MPAI-4 (self) participation (n36)	Mindfulness	1.72 (-11.75, 15.19)	-0.10 (-0.91, 0.70)
	Music	-4.90 (-17.95, 8.16)	0.29 (-0.49, 1.08)
**Informant ratings**		**Mean (SD)**	**[Min, Max]**
BREQ-Relative total (n32)	Mindfulness	52.64 (15.10)	[33.00, 77.00]
	Music	48.14 (11.77)	[32.00, 65.00]
	Audiobooks	47.09 (16.02)	[32.00, 84.00]
MPAI-4 (Informant) total (n30)	Mindfulness	39.44 (13.38)	[19.00, 60.00]
	Music	31.23 (24.32)	[-9.00, 58.00]
	Audiobooks	37.63 (23.20)	[-5.00, 63.00]

The model was adjusted for baseline measure, recruitment location, type of stroke (cortical vs subcortical) and recurrence (first vs recurrent stroke)

BMIPB, BIRT Memory and Information Processing Battery; BREQ, Brain Injury Rehabilitation Trust Regulation of Emotions Questionnaire; FFMQ-SF, Five Faced Mindfulness Questionnaire Short Form; HADS, Hospital Anxiety and Depression Scale; MCQ-30, Metacognitions Questionnaire short form; MoCA, Montreal Cognitive Assessment; Min, minute(s), MPAI-4, Mayo-Portland Adaptability Inventory-4; SART, Sustained Attention Response Test; TEA, Test of every day attention
